# Transcriptional Regulation of Mouse PXR Gene: An Interplay of Transregulatory Factors

**DOI:** 10.1371/journal.pone.0044126

**Published:** 2012-08-28

**Authors:** Sangeeta Kumari, Gauranga Mukhopadhyay, Rakesh K. Tyagi

**Affiliations:** Special Centre for Molecular Medicine, Jawaharlal Nehru University, New Delhi, India; University of Massachusetts Medical, United States of America

## Abstract

Pregnane X Receptor (PXR) is an important ligand-activated nuclear receptor functioning as a ‘master regulator’ of expression of phase I, phase II drug metabolizing enzymes, and members of the drug transporters. PXR is primarily expressed in hepatic tissues and to lesser extent in other non-hepatic tissues both in human and in mice. Although its expression profile is well studied but little is known about the regulatory mechanisms that govern PXR gene expression in these cells. In the present study, we have cloned and characterized over 5 kb (−4963 to +54) region lying upstream of mouse PXR transcription start site. Promoter-reporter assays revealed that the proximal promoter region of up to 1 kb is sufficient to support the expression of PXR in the mouse liver cell lines. It was evident that the 500 bp proximal promoter region contains active binding sites for Ets, Tcf, Ikarose and nuclear factor families of transcription factors. Electrophoretic mobility shift assays demonstrated that the minimal region of 134 bp PXR promoter was able to bind Ets-1 and β-catenin proteins. This result was further confirmed by chromatin immunoprecipitation analysis. In summary, the present study identified a promoter region of mouse PXR gene and the transregulatory factors responsible for PXR promoter activity. The results presented herein are expected to provide important cues to gain further insight into the regulatory mechanisms of PXR function.

## Introduction

Regulation of gene transcription is a fundamental process that is orchestrated by general transcription factors, as well as, ligand-activated transcription factors classified as nuclear receptors. Nuclear receptors function as regulators of gene transcription and they themselves are also regulated by similar processes. It is evident that transcription regulation is dependent on the structure of the promoter region and ever-growing network of interactions on it with co-regulatory proteins. A concept that has developed over the last several years suggests that nuclear receptors and their co-regulators are in a state of dynamics and exert transcriptional control in a combinatorial, coordinated and sequential manner [1]. However, what regulates these nuclear receptors is not as comprehensible and is an area of intensive research pursuit.

The orphan nuclear receptor, Pregnane X Receptor (PXR), is a ligand-modulated transcription factor that protects the body from the harmful effects of foreign or endogenous compounds by activating a set of genes that are involved in drug metabolism and elimination [2], [3]. PXR interacts with a wide spectrum of exogenous ligands such as pesticides, antibiotics, anticancer drugs, as well as endogenous molecules including bile acids and their derivatives, oxysterols, vitamins etc [4]. PXR is primarily expressed in the hepatic tissues and to lower extent in other non-hepatic tissues both in human and in mice [5]. Despite the fact that human PXR and mouse PXR gene transcriptionally respond to important physiologic stimuli and therapeutic drugs [3], [6], till date reports examining regulatory mechanisms that govern PXR gene expression in these cells remain relatively unexplored. A few studies done earlier on characterizing the DNA sequences involved in regulating PXR gene expression focused on the mouse PXR gene [7], [8] but subsequent studies on human PXR gene revealed complexities involved in PXR gene regulation [9]–[11].

In an attempt to understand the molecular mechanisms that regulate PXR gene transcription, we initially cloned and characterized the 5′ UTR of mouse PXR gene. Previously, mouse PXR gene has been shown to possess an HNF4α and farnesoid X receptor (FXR) binding sites in the 5′ UTR and in the intronic regions respectively that regulate its expression [7], [8]. Similarly, in rat, glucocorticoid receptor [GR] has been reported to regulate PXR gene expression both in primary hepatocytes and also in hepatoma cell line [12]. In the present study, we focused on the conserved sequences that lie upstream of, or flank, the transcription start site that appeared to modulate transcription of mouse PXR gene in mouse liver cell lines. Electrophoretic mobility shift and promoter-reporter based transient transfection assays established the involvement of Ets, Tcf, Ikarose and nuclear factor families of transcription factors in regulation of PXR expression.

## Results

### Cloning and functional characterization of mouse PXR promoter

**Figure 1:**
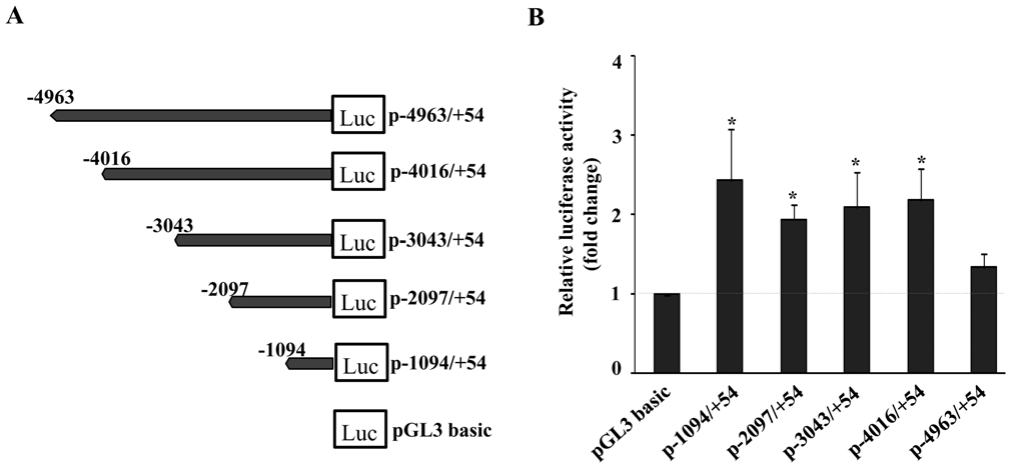
Deletion analysis of ∼5 kb mouse PXR promoter by luciferase reporter gene assays. (**A**) Schematic representation of the mouse PXR promoter and its various deletions constructs. (**B**) Plot showing relative luciferase activities of different constructs. The promoter-less basic luciferase vector and different PXR promoter-luciferase reporter gene constructs were transiently co-transfected into AML-12 cells along with β-galactosidase. After 24 hour of expression period, cell lysate was prepared and luciferase and β-galactosidase activities were determined. Luciferase values were normalized to β-galactosidase and expressed as fold change over the activity of basic luciferase vector. Data represent the mean ± SE of three independent experiments. Asterisks (*) signify luciferase values that differed significantly from the pcDNA transfected cells (P<0.05 in Student's T-test).

To identify the putative regulatory elements in mouse PXR gene, we cloned upto 5 kb region upstream of transcription start site into pGL3 luciferase reporter plasmid and transfected transiently into AML-12 cells to assess their relative reporter gene activities (**Figure 1A**). There was more than two-fold increase in the reporter gene activity when AML-12 cells were transfected with p-1094/+54-Luc construct. However, as the promoter length increased from 1 kb to 5 kb (from −1094 to −4963), the reporter activities were found to decrease sequentially (**Figure 1B**). This result suggests that the cloned 1 kb fragment was sufficient to drive the basal level expression of mouse PXR gene.

**Figure 2:**
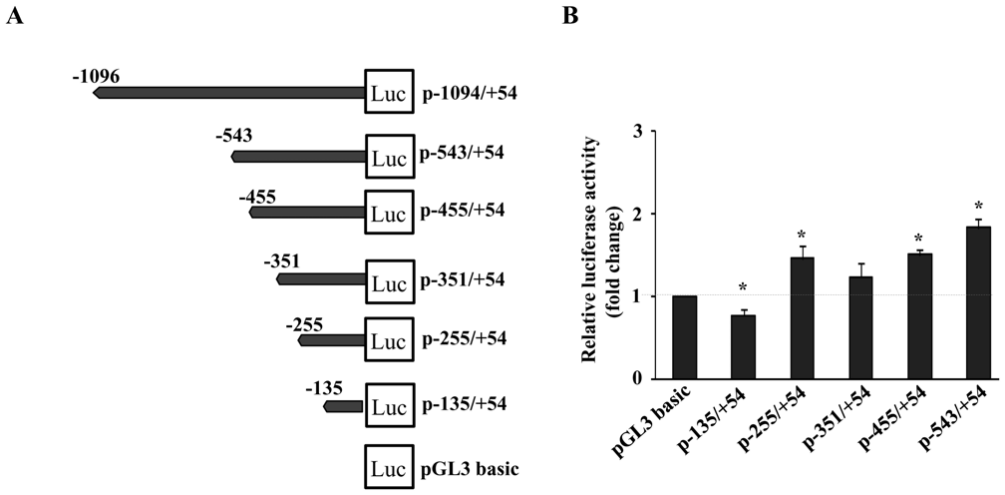
Deletion analysis of 1 kb proximal mouse PXR promoter by luciferase reporter gene assays. (**A**) Schematic representation of the 1 kb mouse PXR proximal promoter and its various serial deletions. The relative positions of different fragments are indicated. (**B**) Different PXR promoter luciferase reporter gene constructs were transiently co-transfected along with β-galactosidase into AML-12 cells. After24 hour of expression period, cell lysate was prepared and luciferase and β-galactosidase activities were determined. Levels of relative luciferase activities of different constructs are shown as fold change over the pGL3 basic vector. Data represent the mean ± SE of three different experiments. Asterisks (*) signify luciferase values that differed significantly from the pcDNA transfected cells (P<0.05 in Student's T-test).

**Figure 3:**
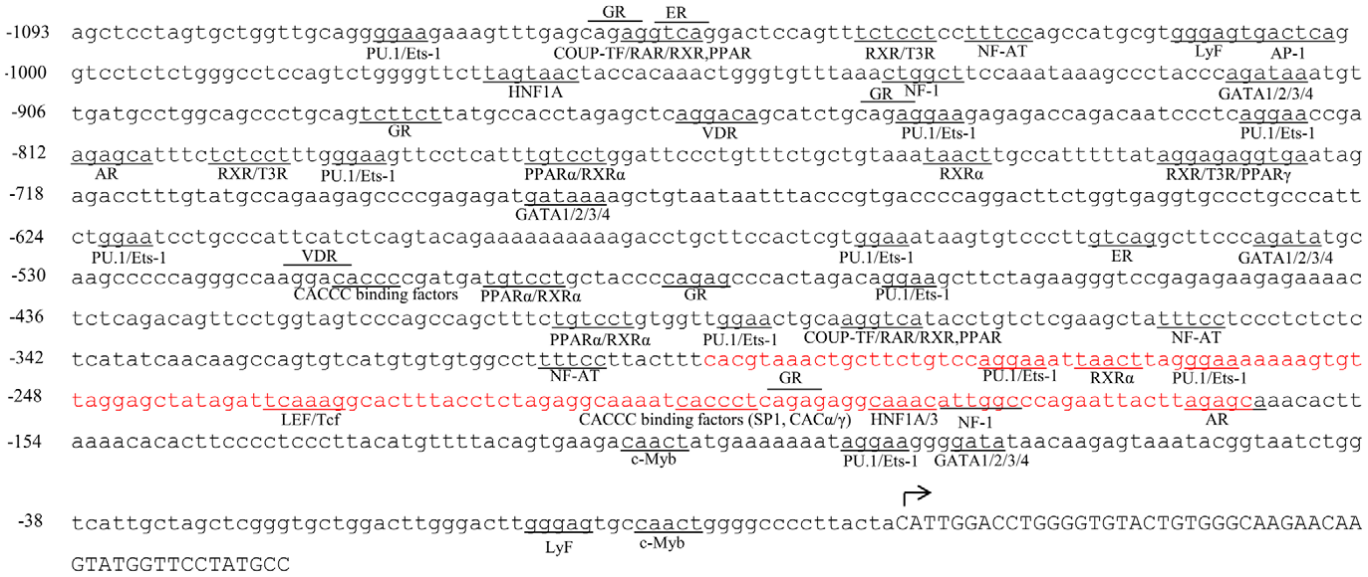
In silico analysis of mouse PXR proximal promoter. The nucleotide sequence of the 5′ flanking genomic DNA of the mouse PXR gene is shown along with consensus sequences for potential DNA-protein binding sites. Transcription initiation site, +1, is denoted by an arrow. PU.1, purine rich box binding element; Ets-1, Ets binding element; GR, glucocorticoid receptor binding element; ER, estrogen receptor binding element; VDR, vitamin D receptor binding element; COUP-TF, chicken ovalbumin upstream promoter binding element; NF-AT, nuclear factor of activated T cells binding element; AP-1, activator protein binding element; HNF1/3/4, hepatocyte nuclear factor binding element; AR, androgen receptor binding element; RXR/RAR, retinoid X/acid receptor binding site; T3R, thyroid hormone receptor binding element; PPAR, peroxisome proliferator-activated receptor binding element; GATA, gata binding element; LEF, lymphoid enhancer factor binding element; LyF, lymphoid factor binding element; c-Myb, myb (myeloblast) binding element; NF-1, nuclear factor binding element. Region marked with red color shows putative binding sites for different transcription factors and this region is further used for EMSA analysis.

Subsequently, to delineate the mouse PXR promoter activity in details, a series of sequential deletion constructs were generated (**Figure 2A**). A deletion of about 605 bp from the 5′ end of 1 kb promoter fragment (p-543/+54-Luc) showed a decrease in the reporter activity compared to p-1094/+54-Luc construct. A further deletion of 88 bp (p-455/+54-Luc) resulted in a decrease in reporter gene activity which continued till deletion upto −351 (p-351/+54-Luc). However, subsequent progressive deletion resulted in first an increase followed by a decrease in luciferase reporter gene activity (**Figure 2B**). These results indicate that positive regulatory elements that control mouse PXR promoter activity in liver cells are likely to be located within the proximal –255 bp region. Taken together, it suggests the presence of multiple putative regulatory elements in the proximal promoter region. Further, *in silico* analysis of the 1 kb promoter revealed presence of several GGAA elements, the consensus binding sequences for the Ets family of transcription factors. Additional search using the Patch search engine of the TRANSFAC database revealed putative sites for both auxiliary/tissue-specific transcription factors such as HNFs, Sp-1, Ets, Tcfs, LyF, and a large number of ligand-activated transcription factors, including GR, PPARα/γ, ERα/β, AR and PR. Nevertheless, at least two putative Ets binding sites and single site for each of CACCC binding factor, LEF and HNF were identified in the region between −297 to −163 bp (**Figure 3**). This suggests that interactions on mouse PXR promoter are complex having binding sites for many regulatory factors, which is consistent with the paradigm of PXR as a master xenobiotic-sensor capable of responding to many different stimuli under different conditions.

### Supershift assays demonstrate presence of Ets-1 and LEF-1/β-catenin in DNA protein complexes

**Figure 4:**
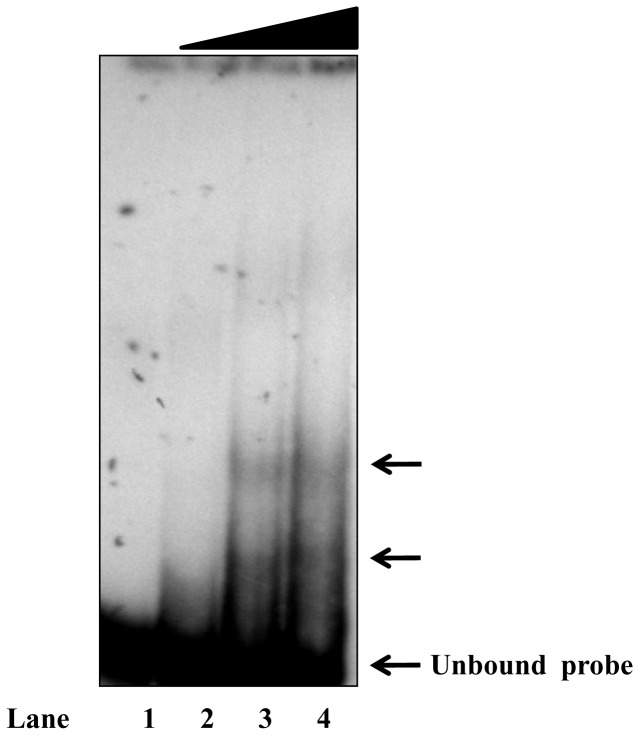
EMSA of mouse PXR proximal promoter. An end-labeled 134 bp promoter fragment (−297/−163) was incubated with AML-12 whole cell lysate. Lane **1**: free probe; Lanes **2, 3** & **4**: binding reactions performed with increasing amount of 3 μg, 6 μg and 12 μg of AML-12 lysate respectively. DNA-protein complexes are shown with arrows.

**Figure 5:**
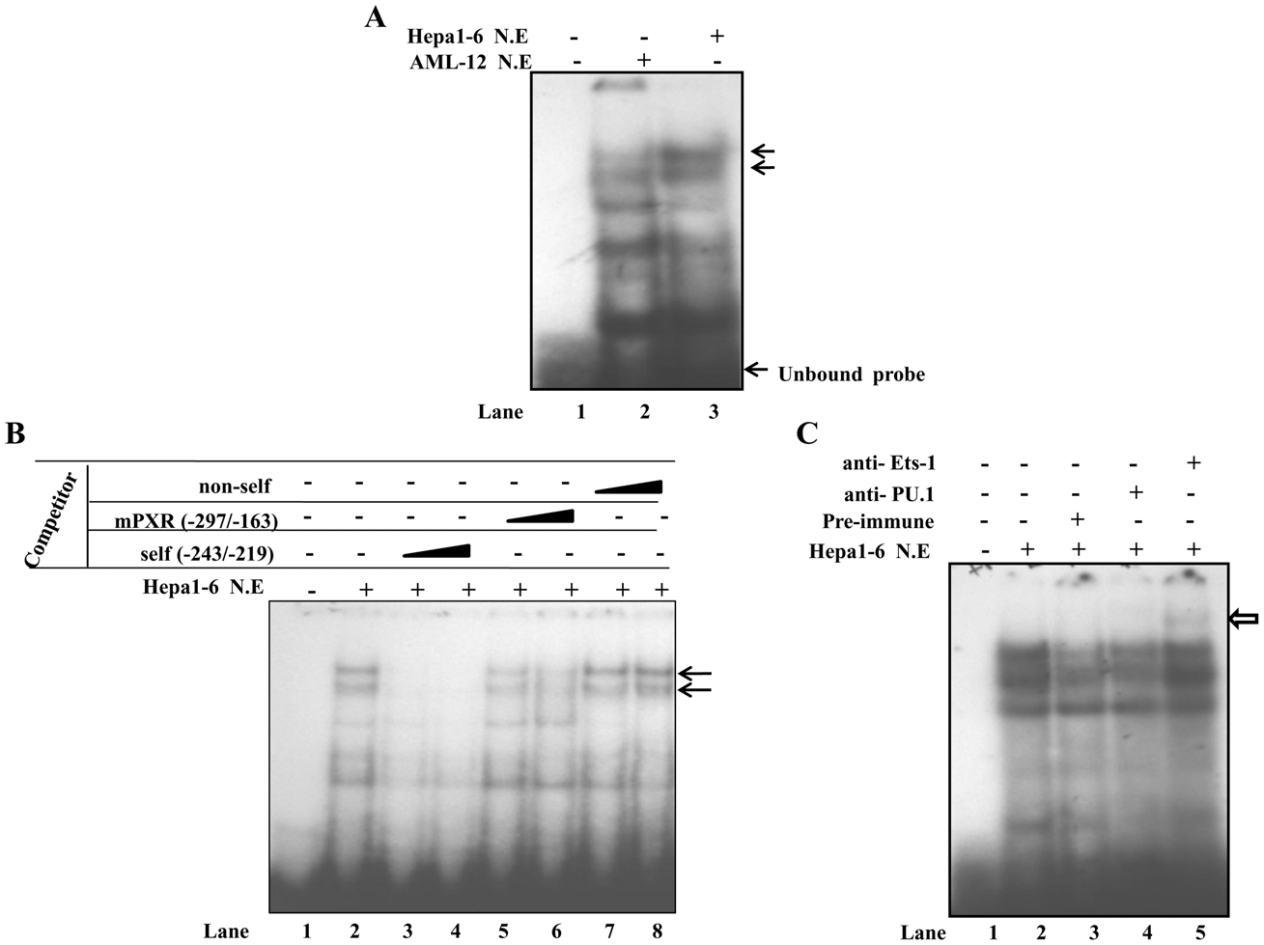
Identification of Ets-1 transcription factor as a protein interacting with −283/−252 oligonucleotide. A ) Radiolabeled DNA probe encoding putative binding sites for Ets family of transcription factors (−283/−252) was incubated with 10–15****μg of Hepa 1–6 and AML-12 nuclear cell lysate. The probe yielded similar band pattern with both the cell lysates. **B**) Competition EMSA was performed using labeled −283/−252 probe, Hepa 1–6 lysate and various competitor oligonucleotides at 50- and 200-fold molar excess. Competitor oligonucleotides used in EMSA are indicated above the figure. Specific bands are shown with arrows. **C**) Radiolabeled −283/−252 oligonucleotide was incubated with Hepa 1–6 nuclear lysate in the presence of either anti-PU.1 or anti-Ets-1 antibodies. DNA-protein complexes were resolved on 5% polyacrylamide gel and supershifted bands are shown with an open arrow. NE =  nuclear extract.

To characterize the transcription factors that interact with the mouse PXR proximal promoter, EMSA was performed using radiolabeled 134 bp PXR promoter fragment (from −297 to −163) and AML-12 cell extract. Incubation of radiolabeled PXR promoter fragment with AML-12 extract yielded two distinctly shifted bands in EMSA (**Figure 4**). Subsequently, two synthetic oligonucleotides containing putative binding sites for Ets and Tcf families of factors respectively were designed from this 134 bp promoter fragment for EMSA (including all the protected and part of the non-protected regions in the PXR promoter fragment tested above). The oligonucleotide PXR (−283/−252) contains putative binding sites for Ets family of proteins while oligonucleotide PXR (−243/−219) corresponds to that of Tcf/LEF family. To test the binding of Ets family of proteins, the oligonucleotide (−283/−252) was radiolabeled with [^32^p] dATP and incubated with equal amounts of AML-12 and Hepa 1–6 nuclear cell extracts separately. As shown in **Figure 5A**, both the extracts yielded similar band patterns of retarded DNA-protein complexes in EMSA. Because of faster growth rate of Hepa 1–6 cells over AML-12 cells, the former was considered for subsequent EMSA. Self-competition experiments with 50 and 200 molar excesses of cold competitors ablated the two slow migrating complexes marked by arrows (**Figure 5B** **, lanes 3 and 4**). Upon competition with ‘−297/−163’ fragment, band intensity of the DNA-protein complex formed was reduced (**lanes 5 and 6**) whereas addition of non-self oligonucleotide neither reduced nor abolished the complex formation even at the highest concentration tested (**lanes 7 and 8**). Further, to substantiate the specificity of binding, supershift assay was performed by incubating radiolabeled probe with Hepa 1–6 cell extract in the presence of pre-immune IgG or antibodies specific for the Ets family of transcription factors, PU.1 or Ets-1. As shown in **Figure 5C**, addition of anti-PU.1 antibody did not show any supershift, however, addition of anti-Ets-1 antibody to the preformed DNA-protein complexes, albeit weak, resulted in the appearance of a supershifted DNA-protein complex. Taken together, these results suggest the involvement of Ets family of proteins, Ets-1, but not PU.1 in the DNA-protein complex formation.

**Figure 6:**
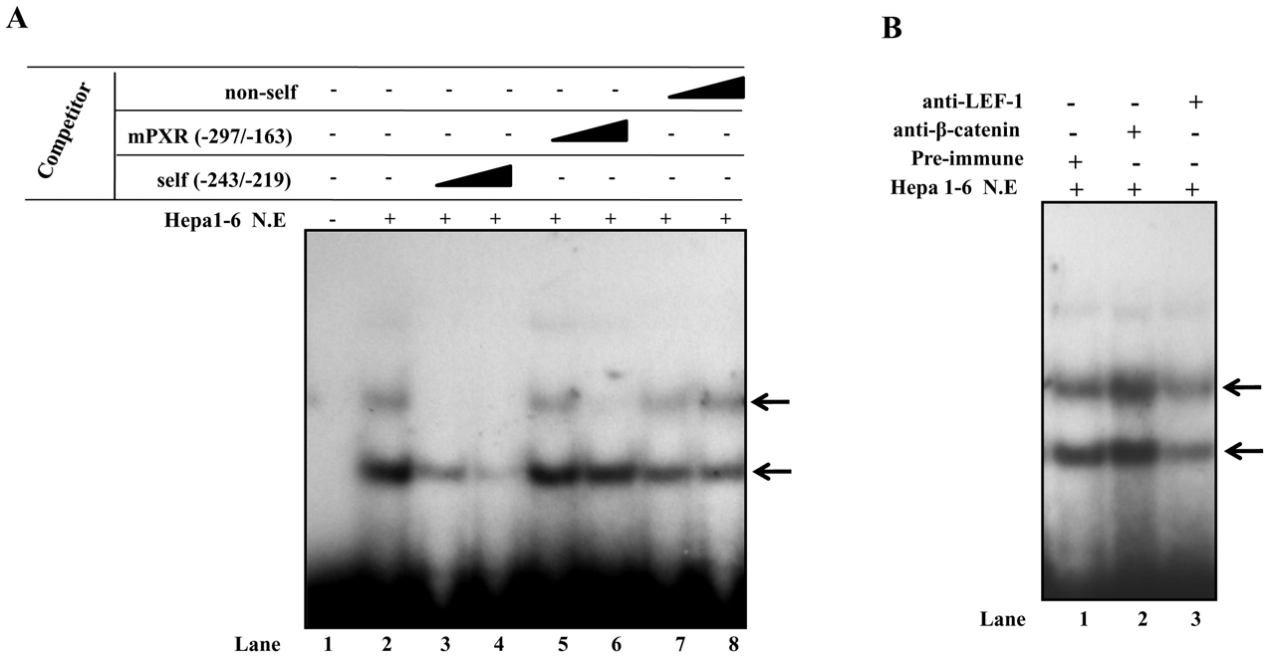
Binding of β-catenin/LEF transcription factors to −243/−219 mouse PXR promoter region. A ) Radiolabeled −243/−219 oligonucleotide was incubated with 15 μg of Hepa 1–6 nuclear lysate (lane 2). For competition experiments, unlabelled −243/−219 mouse PXR (lanes 3 and 4), unlabelled −297/−163 mouse PXR (lanes 5 and 6) and unlabelled non-self oligonucleotides (lanes 7 and 8) were added to the reactions in 50- and 200-fold molar excess. **B**) Antibodies against β-catenin (lane 2), LEF-1(lane 3) or pre-immune IgG (lane 1) were added to the DNA-protein complexes and incubated for an additional 15 minutes at room temperature. Supershifted bands are shown with an open arrow. NE = nuclear extract.

Next, we evaluated oligonucleotide PXR (−243/−219) containing putative Tcf/LEF binding site for protein binding in EMSA. Incubation of radiolabeled probe with Hepa1–6 nuclear extract resulted in specific DNA-protein complex formation (**Figure 6A**). Self-competition with cold oligonucleotide PXR (−243/−219) completely abolished the complex formation (**lanes 3 and 4**) while competition with −297/−163 PXR fragment showed reduction in the protein binding as evident from **lanes 5 and 6**. However, the DNA-protein complex formation was unaffected by the non-self oligonucleotide competitor even at the higher concentration tested (**lanes 7 and 8**). Furthermore, to test the specificity of LEF binding, EMSA was performed either in the presence or in the absence of its interacting partner, β-catenin, in supershift assay. As shown in **Figure 6B**, addition of β-catenin antibody to the pre-formed DNA-protein complex resulted in complex stabilization (as evident by increase in band intensity of complex) while addition of LEF antibody to the preformed DNA-protein complex weakened this interaction as compared to the pre-immune (**lane** **4**). These results suggest the involvement of both LEF-1 and its interacting partner β-catenin in the DNA-protein complex formation at the −243/−219 mouse PXR proximal promoter.

### Mapping of Ets-1 and LEF/β-catenin binding sites on mouse PXR promoter (−297 to −163)

**Figure 7:**
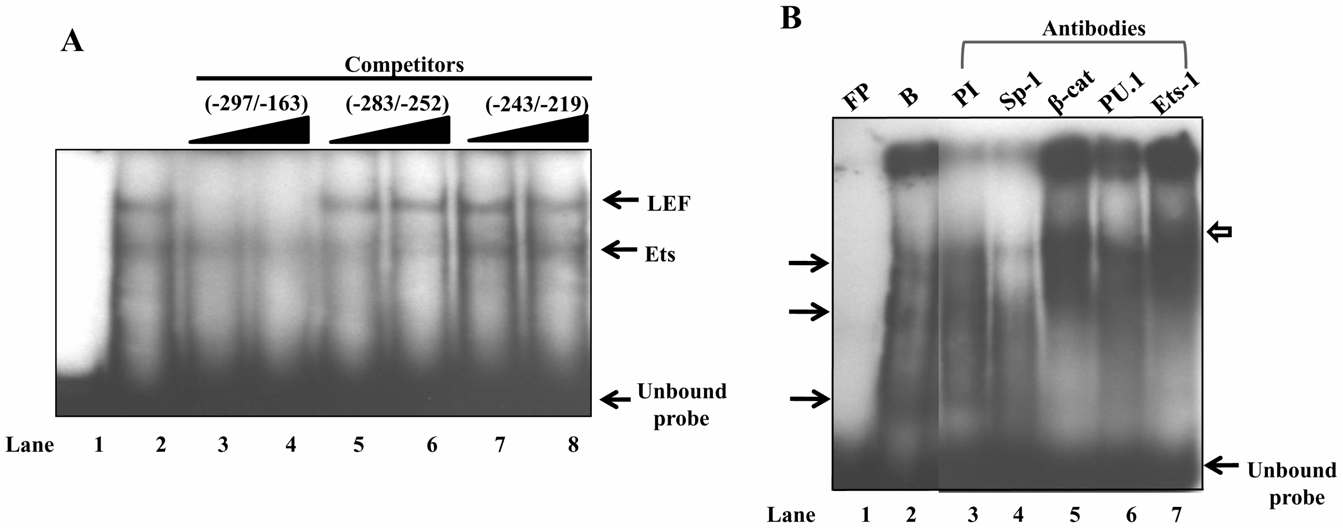
Sequence specific binding of Ets and LEF transcription factors to −297/−163 mouse proximal PXR promoter. A ) A 134 bp (−297 to −163) PCR amplified fragment was radiolabeled and incubated with Hepa 1–6 cell lysate and competition assays were performed with 50- to 100-fold molar excess of unlabelled self-nucleotides (lanes 3 and 4), unlabelled oligonucleotide −283/−252 (lanes 5 and 6) and unlabelled oligonucleotide −243/−219 (lanes 7 and 8). **B**) Supershift EMSA, designed to identify specific protein interactions with −297/−163 radiolabeled probe. The reaction mixtures were incubated with pre-immune (lane 3), Sp-1(lane 4), β-catenin (lane 5), PU.1 (lane 6) and Ets-1(lane 7) antibodies before DNA-protein complexes were subjected to electrophoresis on 5% native PAGE (15 μg of protein/lane). Specific bands are shown with the filled arrows and super-shifted bands are shown with an open arrow.

To substantiate the binding of Ets-1 and LEF/β-catenin onto the 134 bp PXR fragment (from −297 to −163) which encompasses the binding sites for other transcription factors, EMSAs were performed as above. Competition analysis with the unlabeled self-fragment completely abolished the complex formation (**Figure 7** **, lanes 3 and 4**) whereas when oligonucleotide PXR (−283/−252) and oligonucleotide PXR (−243/−219) were used as competitors, partial reductions in intensities were observed in the formation of the faster migrating and slower migrating complexes respectively. These results indicate the presence of Ets like protein in the faster moving DNA-protein complex (**Figure 7A** **, lanes 5 and 6**) and LEF-1/β-catenin like proteins in the slower moving complex (**Figure 7A** **, lanes 7 and 8**). Furthermore, when antibodies against the desired transcription factors that were predicted *in silico* to bind to the PXR promoter were used in supershift assays, only β-catenin and Ets-1 antibodies resulted in the supershifted complex formation (**Figure 7B** **, lanes 5 and 7**) while Sp-1 and PU.1 antibodies did not show any supershift (**Figure 7B** **, lanes 4 and 6**).

### *In vivo* binding of Ets-1 and LEF/β-catenin to mouse PXR promoter

**Figure 8:**
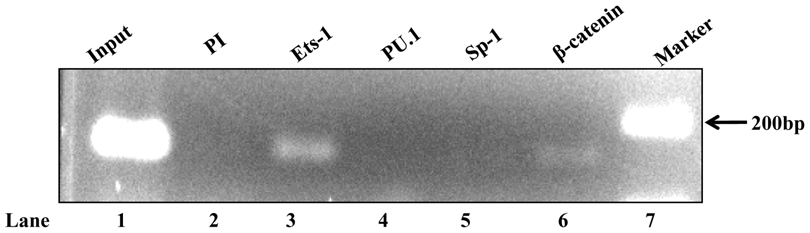
A typical ChIP assay showing binding of Ets-1 and LEF/β-catenin to mouse PXR proximal promoter. Lane** 1** denotes PCR amplification of input DNA; lane** 2** shows PCR amplification of DNA immunoprecipitated using pre-immune serum (control IgG) and lanes** 3 & 6** represent PCR amplification of DNA immunoprecipitated by Ets-1 and β-catenin antibodies. No DNA was immunoprecipitated using PU.1 and Sp-1 antibodies (lanes** 4 & 5**).

Furthermore, to test the *in vivo* binding of Ets-1 to mouse PXR promoter, ChIP assays were performed using Ets-1 and PU.1 antibodies. Immunoprecipitation of the chromatin isolated from Hepa 1–6 cells was followed by PCR using a set of primers specific for 134 bp proximal promoter region of the PXR. Of the two specific antibodies used, only Ets-1 showed distinct binding (**Figure 8** **, lane 3**) to the specified region while PU.1 did not show any binding to the PXR promoter (**lane 4**). This result confirmed *in vivo* binding of the Ets-1 to the mouse PXR proximal promoter. Interestingly, β-catenin antibody had also immunoprecipitated the DNA-bound LEF-protein complex under *in vivo* conditions implying the importance of LEF/β-catenin in mouse PXR gene regulation (**lane 6**). However, no such amplification was observed either with Sp-1 antibody (**lane 5**) or with control pre-immune antibody (**lane 2**) again confirming the specificity of the Ets-1 and LEF/β-catenin binding to the mouse PXR proximal promoter.

### Effect of predicted transcription factors on mouse PXR promoter activity

**Figure 9:**
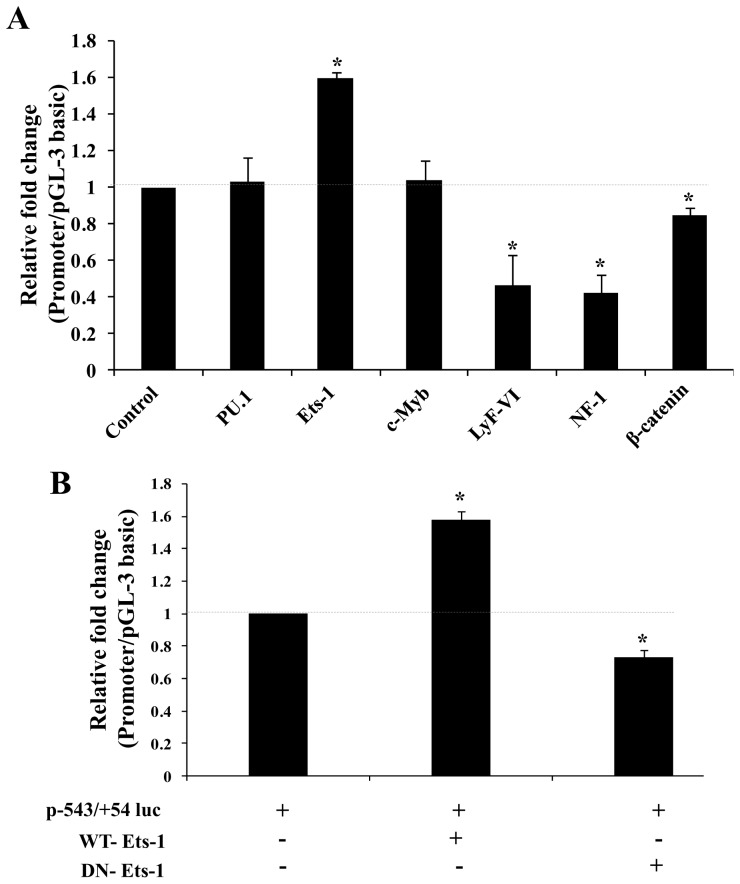
Regulation of mouse PXR proximal promoter by members of different families of transcription factors. A ) The cultured Hepa1-6 cells were co-transfected with p-543/+54-Luc construct and various other expression plasmids encoding for different transcription factors, as indicated in the graph. Following expression period of 24 hours, cell lysates were prepared from the transfected Hepa 1–6 cells and luciferase and β-galactosidase activities were determined. **B**) Hepa 1–6 cells were transiently co-transfected with the p-543/+43-Luc construct together with WT-Ets-1 or DN-Ets-1 expression plasmids. Luciferase values were normalized for transfection efficiency with β-galactosidase values and are expressed as relative fold change with respect to pGL3 basic promoter-less vector. Data represent the mean ± SE of three different experiments. Asterisks (*) signify luciferase values that differed significantly from the pcDNA transfected cells (P<0.05 in Student's T-test).

We transiently co-transfected Hepa 1–6 cells with p-543/+54-Luc construct either alone or concomitantly with different expression plasmids containing genes which were predicted *in silico* for binding to the mouse PXR proximal promoter including Ets, Ikarose, nuclear factor and c-Myb families of proteins. As shown in **Figure 9A**, transfection with Ets-1 expression plasmid transactivated the mouse PXR promoter by 1.6-fold while PU.1 did not show any transactivation. In contrast, dominant negative construct of Ets-1 (DN-Ets-1) markedly diminished the PXR promoter activity (**Figure 9B**). This result is in agreement with *in vitro* (EMSA) and *in vivo* (ChIP) data demonstrating the specificity of Ets-1 towards mouse PXR promoter. However, co-transfection with constitutively active β-catenin construct did not up-regulate the PXR promoter activity (Figure 9A). Nevertheless, transfection with LyF-VI and NF-1 transcription factors strongly reduced the mouse PXR basal promoter activity while c-Myb behaved neutrally with no effect on PXR promoter activity. Overall, these results are consistent with the findings predicting that Ets-1 transactivate the mouse PXR proximal promoter, while Ikarose family (LyF-VI) and Nuclear Factor family (NF-1) of proteins function as transrepressors.

**Table 1:** A list of forward (F) and reverse (R) primers used in the preparation of chimeric PXR promoter-luciferase reporter constructs, EMSA and ChIP analysis.

S. No	Primer name	Sequence (5′–3′)
***Primers used in the generation of 5*** **′** ***deletion PXR promoter reporter constructs***		
1.	−4963 F	GGAATTCACGCGTGTATCAGATCTTCTGGAGCTG
2.	−4016 F	GGAATTCACGCGTTACATCCTGCTCTCTGAGAAC
3.	−3043 F	GGAATTCACGCGTGCTCTATTGAAACTTAACTCTTGC
4.	−2097 F	GGAATTCACGCGTGTCCAGTGATGCACAGCAATG
5	−1094 F	GGAATTCACGCGTAGCTCCTAGTGCTGGTTGC
6.	−543 F	GGAATTCACGCGTCCAGAGCCCACTAGACAG
7.	−455 F	GGAATTCACGCGTGTCCTGTGGTTGGAACTGCA’
8.	−351 F	GGAATTCACGCGTCACGTAAACTGCTTCTGTCCAG’
9	−255 F	GGAATTCACGCGTCTCAGAGAGGCAAACATTGGC
10.	−135 F	GGAATTCACGCGTGAGTAAATACGGTAATCTGGTC’
11.	+54 R	CGGGATCCCTCGAGCCACAGGCATAGGAACCATAC’
***Primers used for EMSA and for ChIP assay***		
12.	−351 F	GGAATTCACGCGTCACGTAAACTGCTTCTGTCCAG
13.	−164 R	CGGGATCCCTCGAGGCTCTAAGTAATTCTGG

## Discussion

PXR is a ligand-modulated nuclear receptor which is responsible for the transcriptional modulation of a large set of cytochrome P450 superfamily members and efflux transporters [5], [13], [14]. However, the intricacies involved in transcriptional regulation of PXR gene itself remain unexplored and warrant further research. Previously, it has been shown that liver enriched factor, HNF4α, can modulate the expression of PXR in hepatocytes through its binding site located in the minimal region of 100 bp upstream of +1 transcription start site [7]. In the present investigation, analysis of distal promoter (5 kb) of mouse PXR gene indicates that the first 1000 bp of the promoter are sufficient to confer maximal liver specific expression of the reporter gene. Based on sequential deletion analysis of the promoter, it is therefore assumed that the mouse PXR expression is mainly driven by the proximal promoter and is affected minimally by the distal region.

The positively modulated region of the PXR promoter (from −297 to −163) identifies putative binding sites for various transcription factors including LEF, HNFs, CACCC binding factors like Sp-1, γCAC1 & 2 along with Ets. Subsequent EMSA and ChIP analysis confirmed the binding of Ets-1 and LEF-1/β-catenin transcription factors to the predicted sites. In this context, we examined the *in vivo* functionality of Ets family of proteins on mouse PXR promoter. In transient transfection expression experiments in Hepa 1–6 cells, Ets-1 activated PXR promoter by 1.6 fold while PU.1, another member of Ets family, did not show any activation. This finding suggests the subfamily specific activation of mouse PXR promoter activity by Ets family of proteins. This is also in accordance with our EMSA results that demonstrated the involvement of Ets-1 in DNA-protein complex formation. On the contrary, Ikarose family and Nuclear Factor family of proteins (LyF-VI and NF-1) behaved as repressors of the mouse PXR promoter activity. Overall, our data supported the notion that PXR promoter is positively modulated by Ets family of proteins and negatively modulated by Ikarose and Nuclear Factor family members and thereby justifying its tight regulation inside the cell. Our inability to observe strong PXR promoter activity in transient transfection assays with different deletion constructs appears to justify that PXR promoter is tightly controlled resulting in maintenance of low levels of PXR inside the cell. This result is also in agreement with the earlier reports that showed its low expression in nearly all mammalian cell lines [15]–[17]. Maintenance of low levels of PXR inside cells may be a normal strategy adapted by the cells during the normal homeostatic control but elevated expression level of PXR may be summoned only during aberrant homeostatic conditions.

The finding that Ets factors participate in transcriptional regulation of PXR gene is consistent with the known functions of Ets proteins. Ets proteins are implicated in the regulation of various important genes [18] and are widely expressed in different tissues and organs, some of which are ubiquitously expressed but others have cell and tissue-specific expression patterns [18], [19]. On the other hand, LEF-1, a HMG box protein, does not possess transcriptional activation potential by itself rather acts as an architectural protein in the assembly of multiprotein enhancer complexes [20]–[22]. LEF is known to perform its function either through β-catenin dependent [20], [23] or independent pathways [24]. Also, β-catenin does not possess any DNA binding domain as such but it binds to DNA in association with Tcf family of proteins as a part of the complex [21], [23]. Though in the context of mouse PXR promoter, the interaction of β-catenin with LEF-1 is evident in EMSA and ChIP analysis, but the constitutively active β-catenin construct did not stimulate the PXR promoter activity in transient transfection assays as Ets-1 did, thereby, suggesting β-catenin to be a part of architectural multiprotein complex which recruit transactivators to up-regulate the promoter activity. Thus, it is apparent that the expression of PXR gene is under tight regulation. A full understanding of the regulatory network of PXR promoter that we have defined in this report may be useful in elucidating the transcriptional perturbations that occur when normal cells become malignant.

## Materials and Methods

### Plasmid constructs

The mammalian expression plasmid for PU.1 (CMV-PU.1) was kindly provided by Dr. Michael Atchison [25]. The expression vectors for LyF-VI, c-Myb and NF-1 were kind gifts from Dr. Stephen Smale (UCLA Microbiology) [26], Dr. Giuseppe Raschella (Ente per le Nuove Technologie, L'Energia EL'Ambiente, Italy) [27] and Dr. N Mermod (University of Lausanne, Switzerland) [28]. The wild type Ets-1 and DN-Ets-1 constructs were kindly provided by Dr. Naofumi Mukaida (Cancer Research Institute, Kanazawa University, Japan) [29]. Mammalian expression plasmid for β-catenin, pCDNA-β-catenin (T41A) was a kind from Dr. Christine Nueveut (INSERM, Paris, France) [30]. The pCMV-β galactosidase expression plasmid was a generous gift from Dr. M. Sharma (Jawaharlal Nehru University, India) [31].

### Cell culture

Cell lines used in the present study Hepa 1–6 (mouse hepatocellular carcinoma cells) and AML-12 (mouse transformed hepatocellular cells) were procured either from National Cell Repository at National Center for Cell Science, Pune, India or directly from American Type Culture Collection (ATCC, Manassas, VA, USA). They were routinely cultured according to ATCC's recommendations in DMEM supplemented with 10% FBS containing 100 µg/ml penicillin, 100 µg/ml streptomycin and 0.25 µg/ml amphoterecin. The cells were maintained at 37^o^C in a humidified incubator in 5% CO_2_ and 95% air atmosphere. All cell culture reagents were obtained from Hyclone (Logan, Utah, USA) or Sigma Chemicals Co. (St. Louis, MO, USA).

### Preparation of whole cell lysate and nuclear extract for electrophoretic mobility shift assays (EMSA)

Whole cell lysate and nuclear extract were prepared from Hepa 1–6 and AML-12 cells. For each extract, 10^6^ cells were harvested by centrifugation and washed twice with icecold phosphate-buffered saline. For preparation of whole cell lysate, mammalian cultured cells from a 100 mm plate were lysed in 100 μl lysis buffer (20 mM Tris pH 7.6, 0.5 mM DTT, 1 mM EDTA, 1 mM PMSF, 0.1% NP 40 and protease inhibitor cocktail) and incubated on ice for 30 minutes with intermittent tapping. After 30 minutes of incubation, 5 M NaCl was added drop-wise to the final concentration of 400 mM with further incubation on ice for 30 minutes. The whole cell lysate was collected by centrifugation at 12,000 rpm for 15 minutes at 4°C. For nuclear extract preparation, cells were resuspended in lysis buffer and incubated for 30 minutes on ice with intermittent tapping. Nuclei were pelleted by centrifugation at 12,000 rpm for 5 minutes and resuspended in same lysis buffer. To this mixture, 5 M NaCl was added to a final concentration of 400 mM and incubated for an additional 30 minutes. Crude nuclear extract was collected by centrifugation at 18,000 rpm for 15 minutes and aliquots were rapidly frozen at −80°C to prevent repeated freezing and thawing. Protein concentrations of the extracts were determined through CB-X protein assay kit (Geno-Technology, MO., USA).

### EMSA

The top and bottom strands of each probe were annealed, and the double-stranded oligonucleotides were end-labeled with [α^32^P]. The. 5 ng of 3′-Klenow end-labeled double stranded probe was incubated with 1–15 µg of protein in 20 µl volume of binding reaction containing 50 mM Tris pH 7.6, 6% glycerol, 1 mM EDTA, 0.1 mM DTT and 1 µg poly dI/dC at room temperature for 20 minutes. Where appropriate, competitor oligonucleotides were included in the binding reaction at 50-, 100- or 200-fold molar excess. For supershift assays, 2–5 µg of each antibody was added to the binding reaction mix before or after the complex formation and further incubated for 15 min at room temperature. The reaction mixtures were electrophoresed in 5% polyacrylamide nondenaturing gel in TBE (89 mM Tris base, 89 mM boric acid and 2 mM EDTA) at room temperature and visualized by autoradiography. The oligonucleotides used either as radio-labeled probes or cold competitors are shown below.

Oligonucleotide PXR (−283/−252): 5′ ACGTCTGTCCAGGAAATTAACTTAGGGAAAAAAAG 3′.

Oligonucleotide PXR (−243/−219): 5′ ACGTCTATAGATTCAAAGGCACTTTACC 3′.

### Generation of 5′ deletion promoter-reporter constructs

For generation of mouse PXR promoter-reporter constructs, a 5 kb fragment encompassing nucleotides from −4963 to +54 of 5′ region of mouse PXR gene was prepared by PCR amplification of mouse genomic DNA obtained from NIH3T3 cells using a forward primer containing *Mlu I* restriction site (5000F) and a reverse primer containing *Xho I* restriction site (+54). The PCR amplified 5 kb product was purified, digested with *Mlu I* and *Xho I* restriction enzymes and cloned directionally into pGL3 basic vector (Promega, Madison, WI, USA). This full-length reporter plasmid was used to generate all subsequent deletion constructs. Reporter genes containing sequentially truncated fragments (nucleotides −4016, −3043, −2097, −1094, −543, −455, −351, −255, −135 to +54) of the PXR 5′-promoter region were prepared by PCR amplification using forward primers containing *Mlu I* restriction site and reverse primers containing *Xho I* restriction site (Table 1). Amplified PCR products were digested with *Mlu I* and *Xho I* and cloned into pGL3 basic firefly luciferase expression plasmid. Region up to 1 kb lying upstream of transcription initiation site is considered as proximal region while region up to 5 kb is considered to be distal.

### Transient transfection and luciferase Assay

Transient DNA transfections in Hepa 1–6 cells were performed either with Escort IV reagent (Sigma) or with lipofectamine (Invitrogen, Carlsbad, CA, USA). Cells were seeded into 12-well plate and transfected with 500 ng of luciferase reporter gene constructs and 125 ng of β-galactosidase reporter gene constructs according to the manufacturer's protocol. Following the transfection period of 10–12 h, cells were incubated for additional 24 h in fresh complete medium. To determine the reporter gene activities, cells were harvested and luciferase and β-galactosidase assays were performed according to the protocol available with the kit (Promega, Madison, WI, USA).

### ChIP assay

Hepa 1–6 cells were used for ChIP analysis. To cross-link DNA and DNA-binding proteins, 10^6^ of Hepa 1–6 cells were fixed with 1% formaldehyde at room temperature for 15 min and reaction was stopped with 2.5 M glycine. The cells were lysed in cell lysis buffer with protease inhibitor cocktail (50 mM Tris pH 7.6, 140 mM NaCl, 1 mM EDTA, 1% Triton X 100, 0.1% sodium deoxycholate and 0.1% SDS). The lysate was sonicated under conditions yielding fragments ranging from 500 to 1000 bp. One-tenth diluted lysate was used for input, and the residual lysate was subjected to immunoprecipitation. Before precipitation, the lysate was precleared at 4°C with protein A-agarose beads (Geno-technology, St. Louis, USA) coated with salmon sperm DNA. The resulting precleared lysate was diluted in immunoprecipitation buffer (1% Triton X-100, 2 mM EDTA p H8, 150 mM NaCl, 20 mM Tris-Cl pH8 with fresh Protease Inhibitors) and was used for overnight immunoprecipitation with 10 μg of each antibody at 4°C. The DNA-protein-antibody complexes were precipitated with protein A-agarose beads, washed, and treated with proteinase K. The DNA was recovered by purification using phenol/chloroform and ethanol precipitation. Next, the DNA was PCR-amplified in 27 cycles (30 s at 94°C, 20 s at 56°C, and 20 s at 72°C). Products of the reaction were analyzed in 2% agarose gel stained with ethidium bromide. Primers used are shown in Table 1.
